# Iron Oxide Nanoparticles Integrated in 3D-Printed Dosage Forms for Advanced Iron Supplementation

**DOI:** 10.1021/acsnanoscienceau.5c00159

**Published:** 2026-02-03

**Authors:** Mac-Kedson Medeiros Salviano Santos, Alexandre Silva Santos, Ariane Pandolfo Silveira, Diego Sousa-Moura, Idejan Padilha Gross, Ingrid Gracielle Martins da Silva, Luis Alexandre Muehlmann, Cesar Koppe Grisolia, Sebastião William Da Silva, Sônia Nair Báo, Marcilio Cunha-Filho, Marcelo Henrique Sousa

**Affiliations:** † Green Nanotechnology Group, 28127 University of Brasilia, CEP 72220-900 Brasilia-DF, Brazil; ‡ Optical Spectroscopy Laboratory, Institute of Physics, University of Brasilia, Brasília-DF 70910-900, Brazil; § Electron Microscopy Laboratory, Cell Biology Department, Institute of Biological Sciences, University of Brasilia, Brasília, Distrito Federal 70910-900, Brazil; ∥ Laboratory of Nanobiotechnology, Department of Genetics and Morphology, Institute of Biological Sciences, University of Brasilia, Brasilia, Distrito Federal 70910-900, Brazil; ⊥ Laboratory of Genetic Toxicology, Department of Genetics and Morphology, Institute of Biological Sciences, University of Brasília, Brasília-DF 70910-900, Brazil; # Laboratory of Food, Drug, and Cosmetics (LTMAC), School of Health Sciences, University of Brasilia, Brasília 70.910-900, DF, Brazil

**Keywords:** iron deficiency, iron supplementation, iron oxide nanoparticles, 3D printlets, fused deposition modeling, hot-melt extrusion

## Abstract

Iron deficiency remains a major global health burden, and conventional oral supplements based on Fe salts are often limited by poor bioavailability and gastrointestinal intolerance. Here, we report an innovative strategy that integrates iron oxide nanoparticles (Fe_3_O_4_ NPs) into three-dimensional (3D) printed tablets (printlets) as advanced oral dosage forms for iron supplementation. Bare and citrate-coated Fe_3_O_4_ NPs were synthesized, dispersed in glycerol, and incorporated into poly­(vinyl alcohol) filaments via hot-melt extrusion, which enabled fabrication of uniform cylindrical tablets through fused deposition modeling (FDM). Structural and spectroscopic analyses confirmed successful incorporation and homogeneous distribution of NPs within the polymeric matrix, while mechanical testing demonstrated that stable glycerol-based dispersions yielded filaments with superior strength and printability compared to powder-based mixtures. Dissolution studies under simulated gastric and intestinal conditions revealed rapid and complete iron release for both formulations at pH 1.0, whereas citrate-functionalized NPs significantly enhanced iron release at pH 6.8, correlating with improved colloidal stabilization of the supernanostructures generated during dissolution. Zebrafish embryo assays further indicated low acute toxicity for both nanoparticle suspensions and dissolved printlets, with citrate coating mitigating hatching delays observed at higher concentrations. Altogether, these results demonstrate that combining iron oxide nanoparticles with FDM-based additive manufacturing enables the production of pharmaceutically relevant and customizable oral dosage forms with improved dissolution behavior and exhibiting low acute toxicity. This platform offers a proof-of-concept toward personalized iron supplementation therapies that address the limitations of conventional formulations.

## Graphical abstract

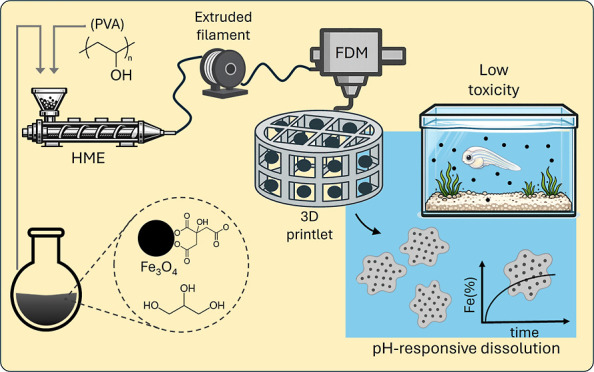

## Introduction

Iron deficiency is one of the most prevalent nutritional deficiencies worldwide, raising significant concerns for health organizations, particularly among at-risk groups such as pregnant women, children, and individuals with chronic conditions.^1^ Although often asymptomatic, untreated iron deficiency can progress to anemia,^2^ leading to impaired cognitive function, dizziness, headaches, weakened immune response, and, in severe cases, hemodynamic instability. Current therapeutic strategies rely mainly on oral^3^ and intravenous supplementation, with the oral route being the most common and safest option.

However, conventional formulations based on Iron salts, typically administrated as tablets or suspensions,^4^ are limited by poor bioavailability and frequent gastrointestinal side effects. In fact, these adverse effects —including nausea, epigastric discomfort, constipation, diarrhea, and metallic taste —are among the leading causes of poor compliance with long-term therapy, often resulting in treatment discontinuation^5,6^ Furthermore, the variable solubility of Iron salts in the gastrointestinal environment, coupled with their susceptibility to oxidation and interaction with dietary components, further compromises absorption efficiency and contribute to inconsistent therapeutic outcomes.^6,7^

In this scenario, nanoformulations–notably those based on iron oxide nanoparticles (NPs) have emerged as a promising strategy to mitigate adverse effects and optimize dosing in iron supplementation.^8,9^ Indeed, their physicochemical stability, high iron content, and biocompatibility make iron oxide NPs particularly attractive for oral supplementation, as they can circumvent the solubility and oxidative degradation issues associated with Iron salts.^10^ Once ingested, these iron oxide NPs can be absorbed by enteric cells through endocytosis mechanisms,^11^ followed by lysosomal dissolution that releases bioavailable iron directly within the cell, minimizing free iron exposure in the gastrointestinal lumen, thereby reducing local irritation.^10^ Despite these advantages, translating these NPs into pharmaceutical dosage forms still faces significant challenges, including the need for dosage adjustments and the design of delivery systems capable of providing enhanced control and personalization.

In this context, 3D printing of pharmaceutical forms stands out as an innovative technology,^12,13^ capable of creating personalized dosage forms through layer-by-layer construction using polymeric matrices with incorporated drugs or supplements.^14^ By adjusting the internal structure of the printed devices, it is possible to modulate their size, shape, dosage, and release profile.^15,16^ This structural flexibility allows the design of systems with tailored dissolution kinetics, site-specific delivery, or even combination therapies within a single unit.^17,18^ Among the available techniques, fused deposition modeling (FDM) is particularly relevant due to its accessibility, cost-effectiveness,^19^ and compatibility with a wide range of polymers.^20,21^ It also enables the incorporation of heat-stable actives, such as iron oxide NPs, directly into extruded filaments, ensuring homogeneous distribution and precise dosing.^22^ Furthermore, FDM supports the production of modified-release tablets and multidrug formulations, offering opportunities to streamline therapeutic regimens and enhance patient adherence.^23^ For iron supplementation, this approach is especially advantageous, providing dosage forms tailored to individual needs and facilitating personalized treatments.^24,25^

Combining iron oxide NPs with FDM-based 3D printing merges the intrinsic benefits of nanoparticle-mediated iron delivery with the manufacturing flexibility of additive manufacturing, paving the way for dosage forms that maximize bioavailability while reducing adverse effects.^17,22^ To date, no studies have reported the use of this combined strategy for developing oral iron supplementation dosage forms.

In this work, we present the development of 3D-printed tablets containing iron oxide nanoparticles (Fe_3_O_4_) as an innovative platform for oral iron supplementation. The approach addresses key limitations of conventional formulations–such as poor bioavailability and gastrointestinal side effects–while overcoming technical challenges related to the homogeneous incorporation of nanoparticles into solid dosage forms.

To this end, Fe_3_O_4_ nanoparticles with distinct surface chemistries (uncoated and citrate-coated) were synthesized, colloidally dispersed in glycerol, and processed via hot-melt extrusion (HME) with poly­(vinyl alcohol) (PVA) to obtain uniform composite filaments. These filaments were subsequently used to fabricate cylindrical tablets by FDM 3D printing (printlets). The resulting printlets were thoroughly evaluated through physicochemical characterization, dissolution testing under simulated gastric and intestinal conditions, and biological safety assessment using the zebrafish (*Danio rerio*) embryo toxicity model. This design enabled a systematic investigation of how nanoparticle surface functionalization and dispersion medium influence filament homogeneity, printability, release kinetics, and favorable preliminary safety profile.

## Materials and Methods

### Materials

Ferric chloride hexahydrate (FeCl_3_·6H_2_O, ≥99%), ferrous chloride tetrahydrate (FeCl_2_·4H_2_O, ≥98%), and sodium citrate tribasic dihydrate (Na_3_C_6_H_5_O_7_·2H_2_O, ≥99%) were obtained from Sigma-Aldrich (St. Louis, MO, USA). Ammonium hydroxide solution (NH_4_OH, 28–30%), hydrochloric acid (HCl, 37%), and acetone (≥99.5%) were supplied by Merck (Darmstadt, Germany). Poly­(vinyl alcohol) (Parteck MXP, 88% hydrolyzed, lot F1952064) and glycerol (≥99.5%, ACS reagent grade) were also obtained from Merck (Darmstadt, Germany). Ultrapure deionized water (resistivity 18.2 MΩ·cm) was produced using a Milli-Q purification system (Millipore, Burlington, MA, USA) and used in all preparations. All reagents and solvents were of analytical grade.

### Elaboration of 3D-Printed Tablets Containing Iron Oxide NPs

The fabrication of 3D-printed iron-release tablets (printlets) involved three sequential stages, as illustrated in Figure 1: (i) synthesis of iron oxide NPs (Fe_3_O_4_), either uncoated (FeP) or citrate-functionalized (FeC); (ii) production of poly­(vinyl alcohol) (PVA)-based composite filaments containing Fe_3_O_4_ NPs via hot-melt extrusion (HME) with glycerol as plasticizer; and (iii) 3D printing of cylindrical tablets by fused deposition modeling (FDM) using the extruded filaments. Each stage was optimized to ensure homogeneous nanoparticle incorporation, precise control of filament geometry, and reproducible tablet architecture, thereby enabling systematic evaluation of physicochemical properties and performance. The procedures for each step are described in detail below.

**Figure 1:**
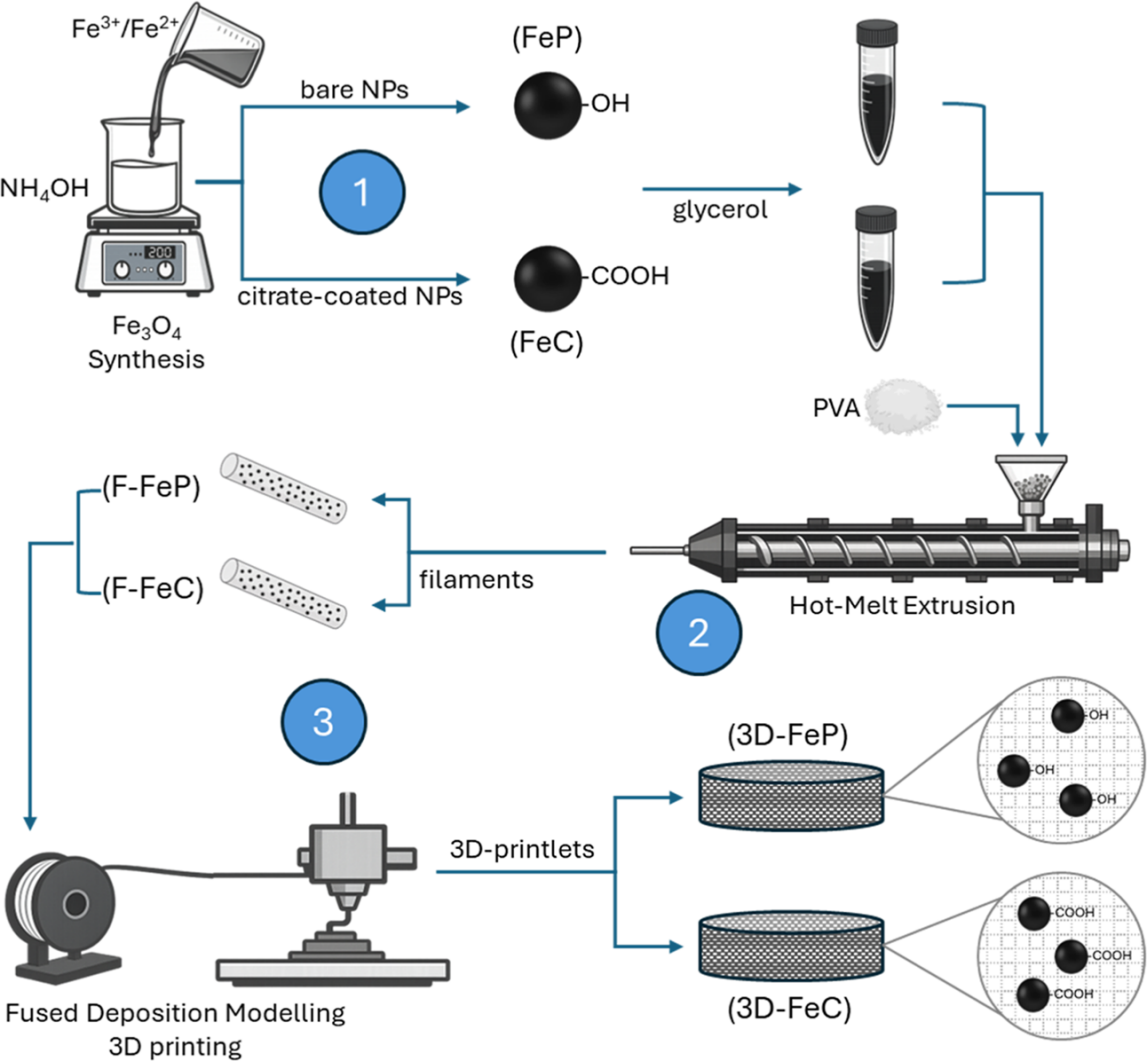
Schematic workflow for the preparation of 3D-printed iron-release tablets (printlets). (1) Synthesis of iron oxide nanoparticles: magnetite (Fe_3_O_4_) was produced via coprecipitation of Fe^2+^ and Fe^3+^ salts in alkaline medium, yielding uncoated (FeP) and citrate-functionalized (FeC) nanoparticles, which were suspended in glycerol. (2) Filament fabrication by hot-melt extrusion (HME): Bare or citrate-coated nanoparticles, dispersed in glycerol, and combined with poly­(vinyl alcohol) (PVA) to produce composite filaments (15 wt % Fe_3_O_4_ nanoparticles, 20 wt % glycerol, 65 wt % PVA) using a corotating twin-screw extruder. (3) 3D printing by fused deposition modeling (FDM): cylindrical printlets (13.55 mm × 4.10 mm, 50% infill, crosshatched pattern) were printed at 220 °C with a 0.4 mm nozzle, generating the printlets 3D-FeP and 3D-FeC, respectively with bare and with citrate-coated Fe_3_O_4_ nanoparticles homogeneously incorporated.

### Synthesis of Iron Oxide NPs

The iron oxide NPs were synthesized via coprecipitation using an adapted protocol previously utilized for the preparation of magnetite (Fe_3_O_4_).^26^ The procedure involved adding 500 mL of an acidic aqueous solution (HCl, pH 1) containing Fe^2+^ (0.25 mol) and Fe^3+^ (0.5 mol) to 2000 mL of NH_4_OH solution (2 mol) under vigorous magnetic stirring. After 1 h of reaction, the precipitate was magnetically separated and thoroughly washed with deionized water until a neutral pH (∼7) was achieved, to yield pristine (or bare) magnetite NPs–sample designated as FeP. A portion of this precipitate also underwent surface functionalization with citrate as adapted from previous work.^27^ For this step, 1 g of FeP was dispersed in 50 mL of an aqueous solution containing 0.7 g of sodium citrate. The mixture was maintained under continuous stirring and heating at 60 °C for 1 h. The pH was adjusted to ∼7, and the excess citrate was removed by washing the product with acetone to yield citrate-coated NPs (designated as FeC). Prior to extrusion, the NPs–either bare (FeP) or citrate-coated (FeC)–were redispersed in glycerol to form stable colloidal suspensions. Part of the nanoparticles were also dried in an oven at 80 °C for characterization purposes and for other tests described in the following sections.

### Filament Production by Hot-Melt Extrusion (HME)

Filaments were produced by HME using PVA as a polymer matrix and the suspensions of glycerol (plasticizer) with dispersed iron oxide NPs (FeP or FeC). The formulations, typically containing iron oxide NPs (15 wt %), glycerol (20 wt %), and PVA (65 wt %), were initially homogenized using a mortar and pestle to ensure uniform distribution of components. The resulting mixtures were then extruded in a corotating twin-screw conical extruder (HAAKE MiniCTW, ThermoScientific, Waltham, MA, USA) equipped with a 1.8 mm die and a filament traction unit (FTR1 model) featuring an automated diameter control system.^28^ Extrusion was conducted at 150 °C with a screw speed of 60 rpm, without material recirculation. The resulting filaments were classified according to their composition as F–FeP (uncoated NPs) and F–FeC (citrate-functionalized NPs).

### Production of 3D-Printlets by Fused Deposition Modeling (FDM)

Cylindrical 3D-printed tablets (printlets) were fabricated via fused deposition modeling (FDM) using the previously extruded filaments. Each printlet presented a diameter of 13.55 ± 0.12 mm, a height of 4.10 ± 0.12 mm, and an average volume of 0.569 ± 0.05 cm^3^ (*n* = 10). The internal architecture was designed with a 50% infill density in a crosshatched pattern, generated in Tinkercad and processed for printing with Slic3r. Printing was performed at 220 °C on a Voolt FDM 3D printer (São Paulo, Brazil) equipped with a 0.4 mm nozzle. Upon fabrication, the printlets were vacuum sealed to preserve their structural integrity prior to physicochemical characterization and performance evaluations. The printlets were named according to the filament used, i.e., 3D-FeP for uncoated NPs and 3D-FeC for citrate-functionalized NPs.

### Characterization of Materials

X-ray powder diffraction (XRD) analyses were performed for sample FeP on a Rigaku Miniflex 600 diffractometer operating at 40 kV and 15 mA. Morphological and energy dispersive spectroscopy (EDS) analyses were performed by scanning electron microscopy (SEM, JSM-7001F, Jeol) and transmission electron microscopy (TEM, JEM-1011, Jeol). For SEM, colloidal samples and cross sections of 3D-printed materials were dried, gold-coated (Leica EM SCD 500), and imaged at 10,000× magnification with 15 kV. For TEM, diluted colloidal suspensions (1:300 v/v) were deposited on Formvar-coated copper grids, dried, and imaged at 30,000× magnification with 80 kV. Fourier transform infrared (FTIR) spectra were acquired on a Bruker Vertex 70 spectrometer equipped with an attenuated total reflectance (ATR) accessory, averaging 96 scans at 4 cm^–1^ resolution, with background collected prior to each measurement. Micro-Raman spectra were obtained using a LabRam HR Evolution spectrometer (HORIBA Scientific) equipped with a confocal microscope, CCD detector, and an 1800 lines mm^–1^ grating, employing a 532 nm diode laser (∼1 mW) focused with a 50× objective. Tensile tests were performed on filaments produced by HME using a universal testing machine (Shimadzu EZ Test, Tokyo, Japan) equipped with a 5 kN load cell, until fracture (*n* = 5). Specimens (60 mm length) were tested with an initial grip separation of 30 mm, a preload force of 1 N, and a crosshead speed of 10 mm min^–1^.

Hydrodynamic diameter (*D*_h_) and polydispersity index (PDI) were determined by dynamic light scattering (DLS), and zeta potential (z) by electrophoretic light scattering (ELS), using a Zetasizer Nano Series (Model 3600, Malvern Instruments). D_h_ and PDI were calculated by the Cumulants method, while z was derived from electrophoretic mobility via the Smoluchowski approximation. pH-dependent measurements were performed with an MPT-2 autotitrator (Malvern Instruments) using standard NaOH and/or HNO_3_ solutions as titrants. Iron content from filaments and printlets (*n* = 10) was determined by atomic absorption spectrometry (AAS) after microwave-assisted digestion in a Multiwave PRO system (Anton Paar). Samples (∼10 mg/250 mL) were digested in 3 mL of H_2_O_2_/HNO_3_/H_2_O (1:1:1, v/v) at 800 W, reaching 100 °C in 2 min and held for 10 min, followed by cooling to 55 °C in 8 min.

### Dissolution Studies

The dissolution tests of printlets were conducted using an Ethik Model 299 apparatus (Nova Etica, São Paulo, Brazil) with 900 mL of medium at 37 °C and an agitation speed of 100 rpm (apparatus 2–rotating paddle).^29^ Phosphate buffer (pH 6.8) and 0.1 mol/L HCl (pH 1.0) were used to simulate intestinal and gastric conditions, respectively. Aliquots (5 mL) were collected and replaced with fresh medium at defined intervals–max 6 h (pH 6.8) or 2 h (pH 1.0). Iron content in the samples (*n* = 5) was quantified by AAS as described in the previous section.

Comparative analysis of the dissolution profiles was performed using dissolution efficiency values determined at 6 h for pH 6.8 (DE6) and at 2 h for pH 1.0 (DE2). Dissolution efficiency was calculated as the area under the dissolution curve up to the specified time point, using the trapezoidal integration method, and expressed as a percentage of the theoretical area corresponding to 100% dissolution over the same time interval^30^ Statistical comparison between formulations was carried out by applying a Student’s *t*-test to the DE6 and DE2 values obtained for the 3D-FeP and 3D-FeC printlets, with a significance level of α = 0.05.

### Fish Embryo Toxicity (FET) Tests

The FET assays were conducted in accordance with OECD Guideline 236 ^31^ (OECD, 2025), with adaptations described by Schulte et al. (2021).^32^ Zebrafish embryos were exposed to bare (FeP) and citrate-coated (FeC) Fe_3_O_4_ NPs, as well as to dispersions obtained from 3D printlets (3D-FeP and 3D-FeC), at concentrations of 0, 0.1, 0.4, 1.6, 6.3, 25.1, and 100 mg/L.

For each treatment, 60 embryos were distributed into three replicates in 96-well microplates (Figure S1). Each well contained 250 μL of test solution (*n* = 20) or water as an internal plate control (*n* = 10), with a single embryo placed per well. Exposure began immediately after fertilization and continued for 96 h in a climate chamber (SL-24, Solab Científica). Embryos and larvae were examined daily under a stereomicroscope at 70× (eggs) or 40× (hatched embryos). Prehatching end points included egg coagulation, otolith formation, developmental delay, eye/body pigmentation, somite formation, heartbeat, oedemas, tail-bud detachment, yolk sac absorption, tail malformation, and hatching. Posthatching evaluations included spinal malformation and loss of equilibrium (embryos lying on their side). All parameters were recorded as present or absent. Additionally, chorions from embryos exposed to samples were further analyzed by SEM to assess pore integrity and nanoparticle agglomeration on their surface. The selection of images for this work was based on the results of embryotoxicity tests (parameter: hatching inhibition).

## Results and Discussion

### Iron Oxide NPs

The X-ray diffraction pattern of sample FeP, presented in Figure 2a, exhibits characteristic reflections consistent with the spinel phase of magnetite–Fe_3_O_4_, as indexed in JCPDS card no. 88–0315. Crystallite size was estimated from the broadening of the (311) diffraction peak using the Scherrer equation, yielding an average value of approximately 8.9 nm. A representative TEM image of the FeP sample, displayed in Figure 2b, showed that Fe_3_O_4_ NPs are nearly spherical. Statistical analysis based on the measurement of approximately 200 particles, assuming a log–normal size distribution, indicated an average particle diameter of 9.7 nm with a standard deviation (σ) of approximately 0.31.

**Figure 2:**
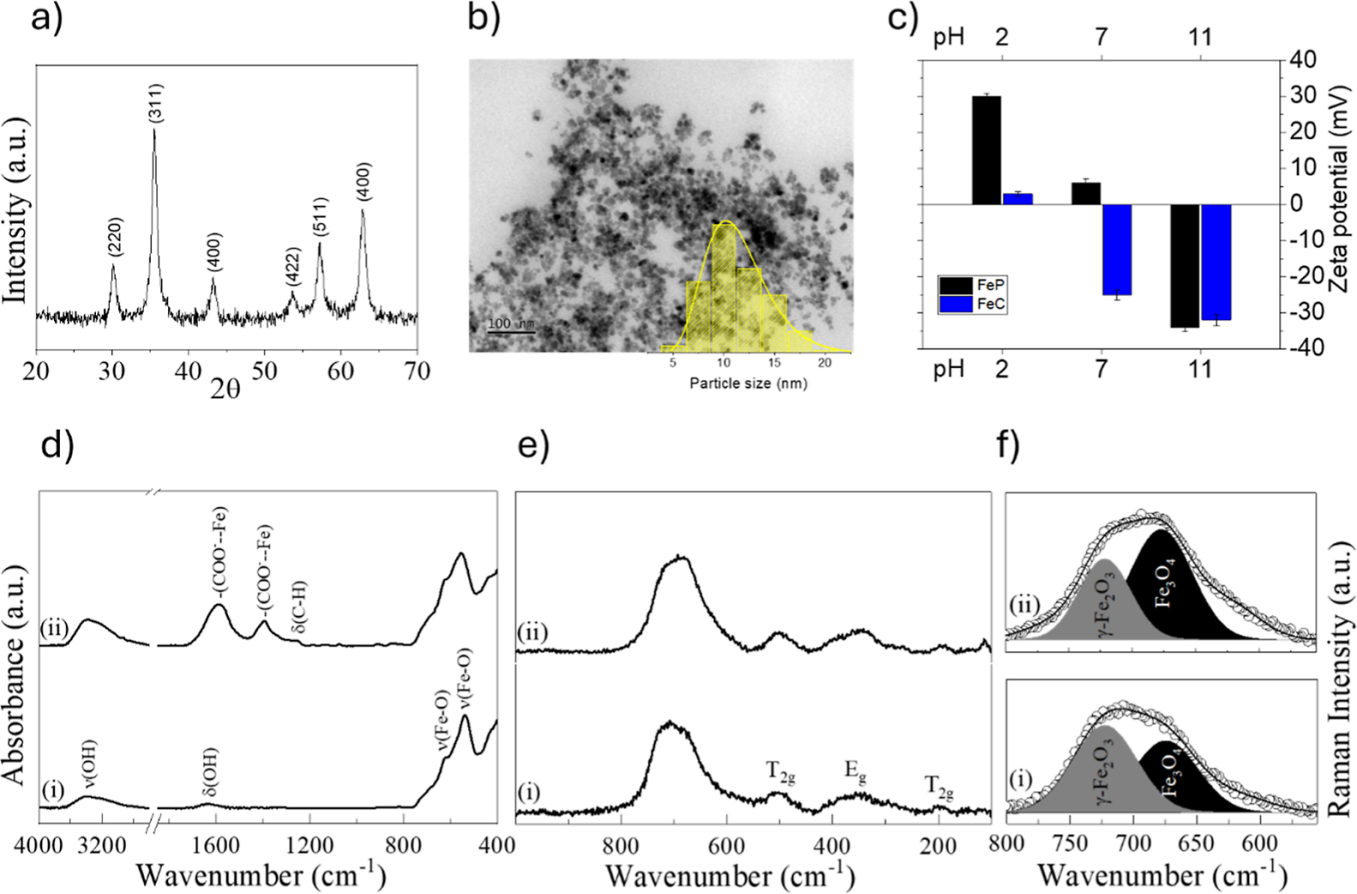
Diffraction pattern (a) and TEM micrograph with 100 nm scale (b) of bare Fe_3_O_4_ nanoparticles (sample FeP)–the inset in (b) shows the particle size distribution histogram. Zeta potential of bare (FeP) and citrate-coated (FeC) Fe_3_O_4_ nanoparticles at different pHs (c). FTIR (d) and Raman (e) spectra of FeP (i) and FeC (ii) Fe_3_O_4_ nanoparticles–magnified view of the 560–800 cm^–1^ Raman region (f).

Figure 2d presents the FTIR spectra of pristine (FeP) and citrate-coated (FeC) Fe_3_O_4_ samples. The FTIR spectrum of bare Fe_3_O_4_ NPs exhibits prominent absorption bands near 600 cm^–1^, which are attributed to the Fe–O stretching vibrations characteristic of the iron oxide lattice. Additionally, a band at ∼1630 cm^–1^, along with a broad absorption region between 3600 and 3100 cm^–1^, corresponds to H–O–H bending and O–H stretching vibrations, respectively. These features indicate the presence of physiosorbed water and surface hydroxyl groups on the NPs.^33^ In the case of citrate-functionalized Fe_3_O_4_ NPs (sample FeC), the presence of bands at 1393 and 1596 cm^–1^ is indicative of coordinated carboxylate groups (−COO–Fe), confirming citrate binding to the NP surface. Additionally, weak absorptions at 2850 and 2930 cm^–1^ are assigned to symmetric stretching modes of CH_2_ and CH_3_ groups, respectively, further supporting the surface adsorption of citrate species. An enhancement in the O–H stretching band near 3370 cm^–1^, when normalized to the 540 cm^–1^ peak, was observed, along with a shift of the Fe–O stretching vibration from 538 to 555 cm^–1^ and a notable broadening of this band. The band at 624 cm^–1^ remained unchanged. Altogether, these spectral features provide strong evidence for the successful surface capping of the magnetite NPs with citrate ligands^33,34^

In Figure 2e, the Raman spectra of the samples FeP and FeC are presented with their respective attributions. Peaks around 198, 352, and 506 cm^–1^ are identified, corresponding to the T_2g_, E_g_, and T_2g_ vibration modes in the oxide structure. The bands associated with magnetite (around 670 cm^–1^) and maghemite (∼720 cm^–1^) indicate that the sample may be partially oxidized (Fe_3_O_4_ → γ-Fe_2_O_3_).^35^ This behavior is more clearly evidenced in the enlarged 560–800 cm^–1^ region, where Gaussian fitting enables separation of the magnetite and maghemite contributions (Figure 2f).

Considering that Raman spectroscopy does not always provide a fully reliable quantification of the oxidation state of magnetite nanoparticles,^36^ classical redox titration using potassium dichromate (dichromatometry) was performed to quantitatively determine the Fe^2+^ and Fe^3+^ contents and, consequently, to estimate the degree of Fe_3_O_4_ → γ-Fe_2_O_3_ oxidation in the nanoparticles. The oxidation degree determined from Fe^2+^/Fe^3+^ quantification for bare (FeP) and citrate-coated (FeC) Fe_3_O_4_ nanoparticles, respectively, (at different stages of the study) was found to be 13.0 ± 0.7% and 9.5 ± 0.5% (after synthesis), 14.8 ± 0.5% and 11.3 ± 0.4% (after hot-melt extrusion and fused deposition modeling), and 14.2 ± 0.7% and 11.8 ± 0.8% (after tablet dissolution in water). Notably, no significant increase in the oxidation degree was observed across the different processing steps, despite exposure to elevated temperatures during hot-melt extrusion and to aqueous environments during dissolution testing. In all cases, the citrate-coated nanoparticles consistently exhibited a lower oxidation degree compared to the bare counterparts.

The lack of significant evolution in the oxidation degree across the different processing stages can be attributed to the combined stabilizing effects of surface functionalization and polymer encapsulation. Citrate functionalization is known to mitigate oxidation and favor preservation of the magnetite phase, enhancing chemical stability from the synthesis stage onward.^34,37^ During hot-melt extrusion and fused deposition modeling, both bare and citrate-coated nanoparticles become embedded within the polymer matrix, which provides an additional physical barrier that limits exposure of the iron oxide surface to oxidative and aqueous environments. Polymer coatings such as PVA have been reported to shield iron oxide cores and mitigate oxidation under harsh chemical or physiological conditions.^38^ Consequently, no measurable increase in oxidation was observed after extrusion or dissolution, while the citrate-coated nanoparticles consistently exhibited a lower overall oxidation degree. Importantly, no significant evolution of the oxidation degree was observed across the different processing steps, and all formulations were processed under identical conditions, supporting the validity of the comparative conclusions regarding iron release and toxicity trends.

The zeta potential (ζ) values of the bare and citrate-coated NPs, evaluated at different pHs, are shown in Figure 2c. The FeP sample reveals NPs with a positively charged surface at pH 2 (∼+ 30 mV), near-zero surface charge at neutral pH, and increasingly negative charge under alkaline conditions, reaching approximately −30 mV at pH 11. This behavior is related to the amphiprotic nature of the pristine surface, which varies according to the pH of the medium–the sites on the surface of the bare iron oxide NPs predominantly are protonated (≡Fe–OH_2_^+^) under acidic conditions, neutral (≡Fe–OH) at intermediate pH, and deprotonated (≡Fe–O^–^) in alkaline environments.^26,39^ Figure 2c further demonstrates that the zeta potential of citrate-coated NPs (sample FeC) exhibited a strong dependence on pH, ranging from approximately +5 mV to −33 mV as the pH increases from 2 to 11. This behavior can be attributed to the progressive deprotonation of surface-bound carboxylic acid groups from the adsorbed citrate molecules as the pH increased (≡Fe–COOH → ≡Fe–COO^–^), leading to a higher density of negatively charged sites and, consequently, more negative zeta potential values.^40^

### HME Filaments

Glycerol-based colloidal suspensions containing bare (FeP) or citrate-coated (FeC) iron oxide NPs were mixed with PVA and extruded by HME to produce, respectively, the filaments F–FeP and F–FeC, which were used to produce the printed tablets and for dissolution and toxicity assays. Preliminary tests showed that when dried NPs were physically mixed with glycerol and PVA (rather than incorporated as glycerol-based dispersions), the resulting slurry exhibited poor homogeneity. These physical mixtures were also evaluated for filament production. In this case, dried powders of FeP and FeC, mixed with PVA and glycerol using a mortar and pestle, were extruded under the same conditions and proportions, yielding the F–FeP* and F–FeC* samples. However, as described below, these filaments were of inferior quality compared to those produced with the stable glycerol-based colloidal suspensions of NPs. This outcome led us to adopt the methodology based on glycerol-based suspensions for filament fabrication and subsequent printlet production.

Indeed, the stress–strain curves of the polymeric filaments incorporating iron oxide NPs (Figure 3a) showed a characteristic ductile behavior, beginning with an initial elastic region, followed by plastic deformation up to a maximum stress point, after which a sharp fracture occurs.

**Figure 3:**
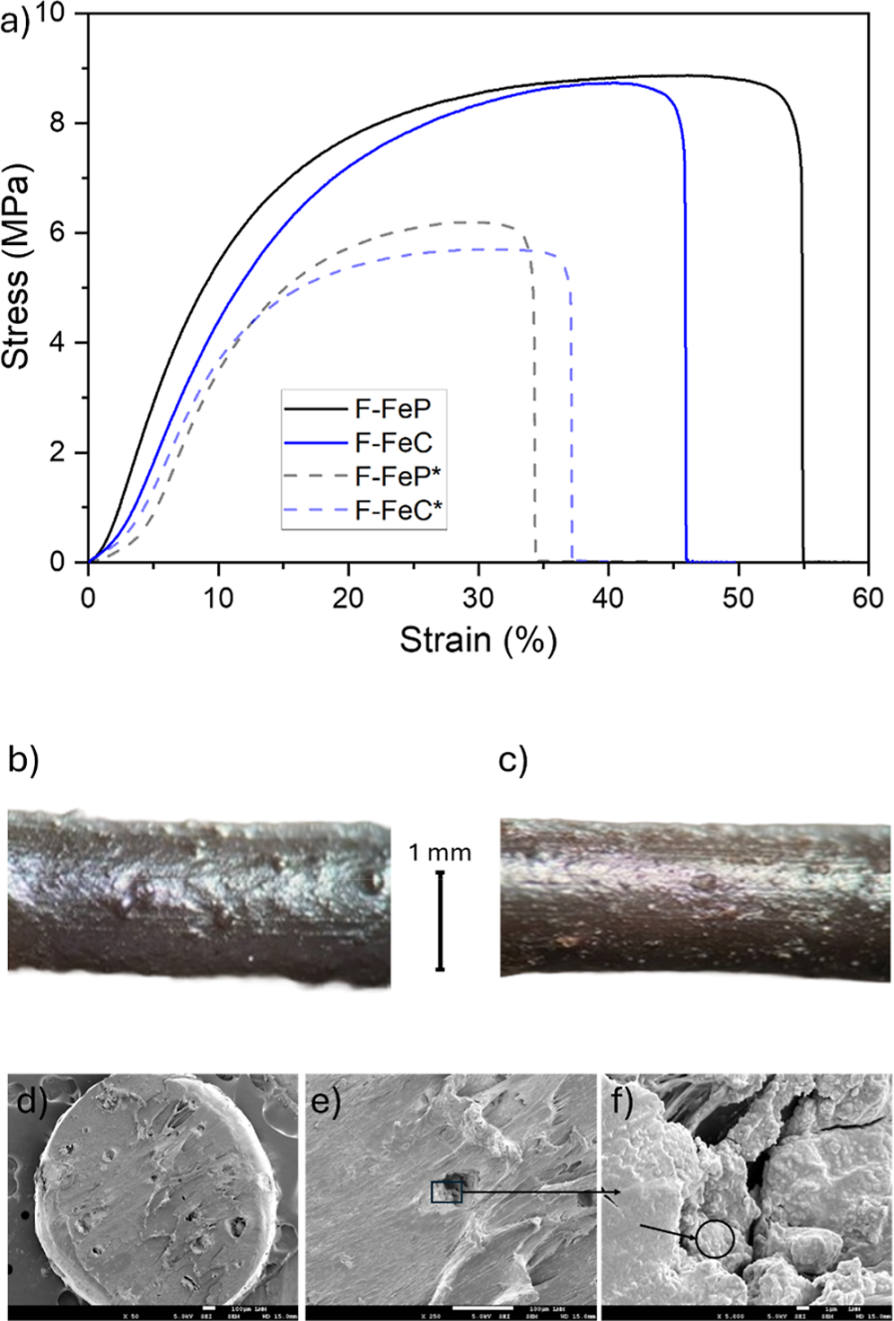
Typical stress–strain curves (a) of extruded filaments containing bare and citrate-coated Fe_3_O_4_ nanoparticles prepared from stable glycerol suspensions (F–FeP and F–FeC–solid lines) and from physical mixture containing dried nanoparticle powders (F–FeP* and F–FeC*–dashed lines). Visual aspect of typical F–FeP (b) and F–FeC (c) filaments. Representative SEM images of a cross-section of F–FeC filament used for tablet printing at 50× (d), 250× (e), and 5000× (f) magnification, showing regions with incorporation of Fe_3_O_4_ nanoparticles. The presence of iron was confirmed by EDS analysis (Figure S2a) of the selected area shown in (f).

Filaments prepared from stable glycerol-based suspensions of Fe_3_O_4_ NPs (F–FeP and F–FeC–solid lines) demonstrated markedly superior mechanical properties compared to those from dried NP powders (F–FeP* and F–FeC*–dashed lines). The uncoated NP formulation (F–FeP) showed the highest tensile strength (approximately 9 MPa) and elongation at break exceeding 50%, indicating enhanced toughness and effective interfacial interaction between the NPs and the PVA matrix,^41,42^ probably associated with the strong hydrogen bonds with the nanoparticle’s surface.

In contrast, the sample formulated with citrate-coated NPs exhibited slightly lower mechanical strength and ductility, possibly due to the reduced affinity between the modified surface and the polymer chains, which may limit stress transfer at the interface. Filaments from dried powders, on the other hand, regardless of surface modification (dashed curves), led to a substantial decrease in both tensile strength and elongation, likely associated with poor dispersion and NP agglomeration, which could act as stress concentration points and promote premature failure.^43^ As summarized in Table 1, the filaments produced from stable suspensions exhibit higher ultimate strength, greater elongation at break, and comparable or higher Young’s modulus than dried-powder formulations.

**Table 1:** - Mechanical Properties Obtained From Stress–Strain Curves, Including Ultimate Strength, Elongation at Break, and Young’s Modulus for Different HME Filament Formulations

sample	ultimate strength (MPa)	elongation at break (%)	Young’s modulus (MPa)
FeP	6.21 ± 0.03	34.6 ± 1.6	50.9 ± 0.3
FeC	5.48 ± 0.22	37.9 ± 5.2	43.4 ± 4.3
F–FeP	9.25 ± 0.41	51.6 ± 8.8	53.3 ± 3.7
F–FeC	8.94 ± 0.32	41.1 ± 4.3	56.8 ± 6.8

These findings underscore a possible critical role of NP dispersion and interfacial compatibility in tailoring the mechanical behavior of extruded nanocomposite filaments. Although citrate coating typically improves NP dispersion in aqueous media through electrostatic stabilization^34^ it does not significantly enhance interfacial adhesion with the PVA matrix.^44^ Conversely, glycerol proved critical, acting as both dispersant and plasticizer, leading to more uniform NP distribution and enhanced mechanical performance.^45,46^ As a result, filaments prepared with glycerol-based stable suspensions (F–FeP and F–FeC) exhibited higher tensile strength and elongation at break. Notably, only filaments from stable suspensions (F–FeP and F–FeC) exceeded the minimum tensile strength threshold (8.5 MPa) required for reliable 3D printing, whereas dried-powder based filaments frequently showed feeding-related failures. Based on these results, F–FeP and F–FeC were selected for 3D tablet fabrication, biological testing, and dissolution studies.

As shown in Figure 3b,c, extrusion of bare and citrate-coated Fe_3_O_4_ NPs yielded uniform cylindrical filaments with diameters of approximately ∼1.7 mm and a consistent black/brown coloration across the material, suggesting a homogeneous distribution of NPs throughout the polymer matrix. SEM analysis (Figure 3d) revealed filaments with a relatively uniform and rough surface morphology, characterized by the presence of numerous pores. At higher magnification (Figure 3e,f), SEM micrographs with EDS mapping (Figure S2a) confirmed the presence of iron oxide nanoparticles embedded within or adhered to the polymeric matrix. Iron content analysis by AAS across different segments of the extruded filaments demonstrated a homogeneous distribution of Fe within the polymer matrix, with average Fe contents of 8.3 and 9.2 wt % for F–FeP and F–FeC, respectively. Considering the theoretical Fe loading of 15 wt %, these values indicate that approximately 55–61 wt % of the expected metallic content was retained after the extrusion and FDM processing steps. This discrepancy does not arise from inhomogeneous nanoparticle incorporation or analytical variability, but rather from material losses inherent to the multistep processing route employed in this work. Specifically, partial iron loss is expected during HME and FDM, where viscous, nanoparticle-rich formulations may partially adhere to the internal surfaces of the extruder barrel, screws, die, and filament traction system. Similar phenomena have been reported for nanoparticle-filled polymeric systems processed by HME and FDM, particularly at high filler loadings.^47^

### 3D Printlets

The 3D-printlets produced from HME filaments containing either bare Fe_3_O_4_ (3D-FeP) or citrate-coated Fe_3_O_4_ NPs (3D-FeC) displayed a uniform black/brown coloration and consistent layered architecture (Figure 4a,b). SEM images of the fractured cross-section reveal the characteristic filamentary and lamellar morphology resulting from the extrusion and FDM processes, with flow-oriented features of the polymer matrix visible across the section (Figure 4c,d). At higher magnification, SEM micrographs and EDS mapping (Figures 4e and S2b) confirmed the presence of Fe_3_O_4_ NPs both adhered to the surface and embedded within the interconnected pores of the polymer network. Quantification of iron by AAS in different sections of the 3D-printlets demonstrated a uniform distribution of Fe within the polymer matrix. The average iron content was 7.1 wt % for the 3D-FeP formulation and 6.8 wt % for the 3D-FeC formulation, with no significant variation between segments of the same printlet. Although the measured values were slightly lower than the theoretical loading of 15 wt %, the findings demonstrate that iron was effectively incorporated and homogeneously dispersed within the polymeric matrix. This outcome suggests that both extrusion and FDM printing preserved the metallic content, despite minor losses likely related to thermal and mechanical stresses during processing.

**Figure 4:**
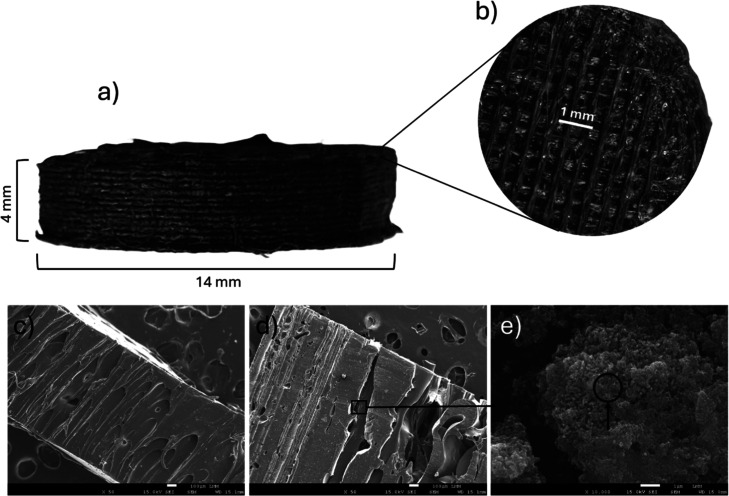
Representative photograph of a typical 3D-printed tablet containing citrate-coated Fe_3_O_4_ nanoparticles (3D-FeC) (a) and magnified view (b) highlighting the braided-like pattern characteristic of the printing process. SEM images (50×) of surface cross-section (c) and interior cross-section (d) of tablet. In (e), a higher magnification (10.000×) reveals the presence of Fe_3_O_4_ nanoparticles embedded within the polymeric matrix, confirmed by the iron signal observed in the EDS analysis of the selected region (Figure S2b).

### Dissolution Studies

Dissolution testing of iron-release printlets serves as a key indicator of dosage form quality, efficacy, and batch-to-batch consistency. The resulting dissolution profiles provide essential information on iron release kinetics, supporting predictions of bioavailability and therapeutic performance.^48^ Figure 5 presents the dissolution profiles–dissolved iron versus time–in simulated intestinal and gastric fluids for printlets produced from HME filaments containing bare and citrate-capped Fe_3_O_4_ NPs, i.e., 3D-FeP and 3D-FeC samples, respectively.

**Figure 5:**
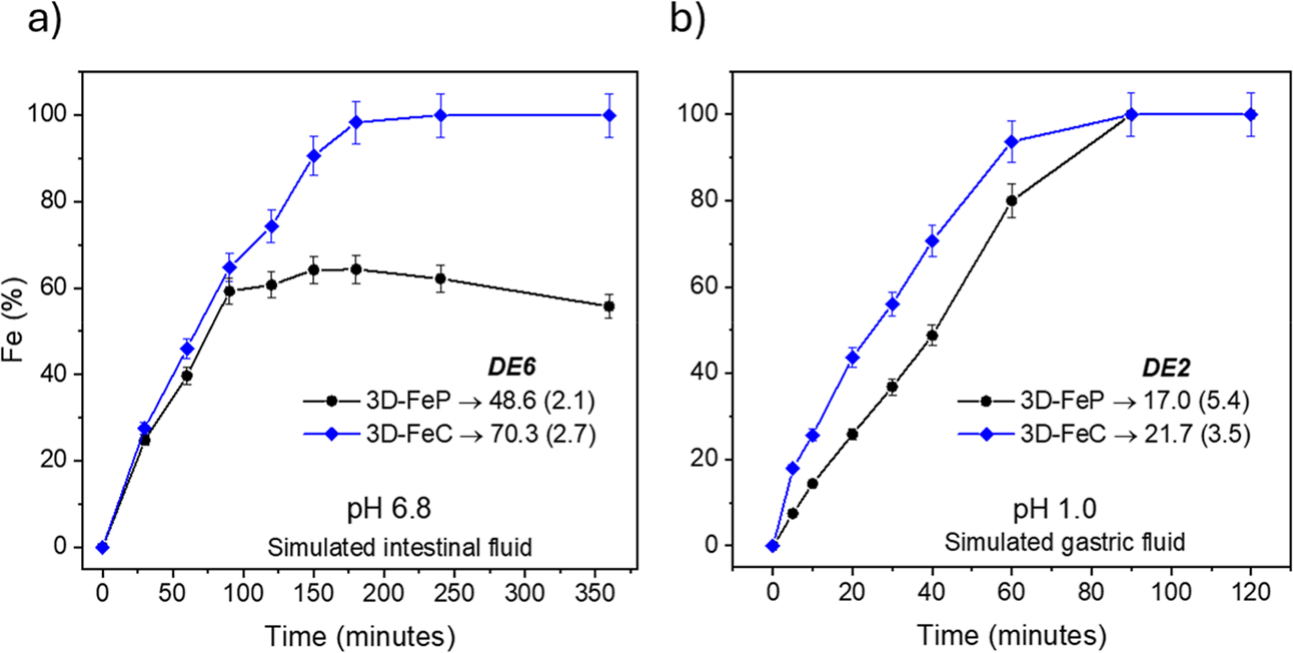
Dissolution profiles of 3D-FeP (black) and 3D-FeC (blue) printlets, containing respectively bare and citrate-coated Fe_3_O_4_ nanoparticles, under simulated conditions of (a) intestinal fluid pH (6.8) and (b) gastric fluid pH (1.0). The values are expressed as mean ± SD. Additionally, mean values of the dissolution efficiency at 2 and 6 h (DE2 and DE6, respectively) with SD in parentheses are presented.

The dissolution profiles for both types of printlets in the different simulated media generally exhibit an initial rapid release phase (burst effect), followed by a slower dissolution rate until reaching equilibrium at a certain time point (Figure 5). However, the dissolution rate varies depending on the medium.

While the dissolution profiles of the 3D-FeP and 3D-FeC samples are equivalent in acidic media, with complete iron dissolution in the medium within 90 min, in enteric media, printlets containing bare NPs are limited to an iron dissolution of approximately 50%. In contrast, in enteric media, printlets containing coated NPs are capable of dispersing nearly 100% of the iron in 150 min (Figure 5). These results are quantitatively supported by the DE analysis, i.e., at pH 6.8, the DE6 values were 48.6 for 3D-FeP and 70.3 for 3D-FeC, confirming the superior extent of iron release under intestinal conditions for the citrate-coated NPs (*p* = 0.0005), In acidic medium (pH 1.0), although 3D-FeC exhibited a slightly higher DE2 value of 21.7 compared with 3D-FeP with 17, this difference was not statistically significant (*p* = 0.202), indicating that both formulations behave equivalently in terms of release efficiency under gastric conditions.

Previous studies have consistently shown that PVA dissolves across a wide pH range, but with kinetics strongly influenced by formulation architecture and polymer properties. Wei et al.^49^ demonstrated that 3D-printed PVA tablets containing carvedilol and haloperidol achieved complete drug release in ∼45 min at both pH 2.0 and 6.8, confirming that PVA enables drug liberation under gastric and intestinal conditions. Similarly, Couţi et al.^50^ highlighted that PVA is a nonionic, pH-independent excipient, capable of dissolving in both acidic and neutral environments, which explains its frequent selection for FDM-based dosage forms intended for rapid or sustained release.

Therefore, such a divergence observed for bare NPs in the enteric medium underscores that while PVA itself is soluble across pH, the nanoparticle surface chemistry probably modulates the dissolution mechanism in neutral/basic environments, an effect that probably emerges from the nature of the NP-polymer interactions.

The analysis of the solution containing the dissolved printlets by TEM revealed the presence of colloidal nanoparticle clusters (supernanoparticles), apparently formed by the disintegration of the tablet into multinucleated structures of iron oxide NPs embedded within polymeric portions.^51^ This observation can be corroborated by zeta potential analyses and by measuring the hydrodynamic diameter of these dispersions as a function of pH (Figure 6). For slurries resulting from the dissolution of tablets composed of both bare and citrate-coated NPs, the zeta potential increases in magnitude as the pH rises from 2 to 12–shifting from slightly positive values to around −10 mV for the 3D-FeP sample and to approximately −20 mV for the 3D-FeC sample.

**Figure 6:**
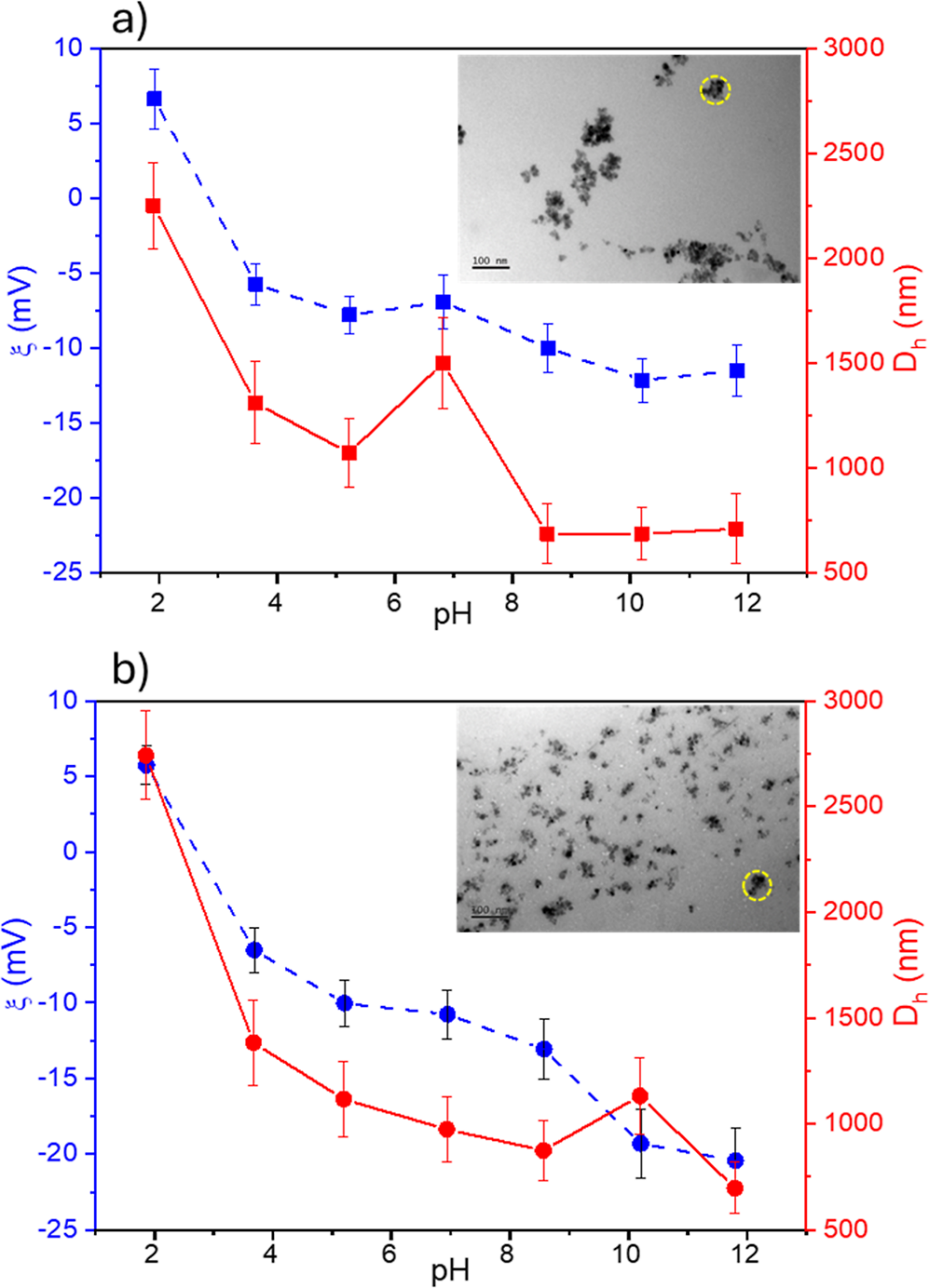
Hydrodynamic size (Dh) and zeta potential (ζ) as a function of pH for solutions containing the dissolved printlets 3D-FeP (a) and 3D-FeC (b) containing respectively bare and citrate-coated Fe_3_O_4_ nanoparticles. The values are expressed as mean ± SD. Insets show representative TEM micrographs of the colloidal nanoparticle clusters (supernanoparticles) formed upon dissolution of the printlets, as highlighted by the circles.

These findings suggest that the surface charge profile of these supernanostructures results from the combined contributions of both constituents, namely the PVA and the Fe_3_O_4_ NPs.^52,53^ Even though PVA does not possess strong ionizable groups that could directly account for a pronounced variation in zeta potential with pH, several well-established mechanisms explain why the zeta potential of PVA-coated or PVA-containing particles tends to become more negative as the pH increases. First, hydroxyl moieties along the PVA backbone can specifically adsorb hydroxide ions from the aqueous medium at higher pH values, inducing a net negative charge at the slipping plane. Second, the underlying particle substrate–such as metal oxides–often presents surface hydroxyl groups (≡M–OH) that can undergo protonation/deprotonation, thereby modulating the overall surface charge, even when partially masked by the PVA layer.

Third, the adsorbed PVA chains may shift the slipping plane and reorganize the electrical double layer, thereby promoting electrosteric effects that enhance the measured negative potential. Additionally, residual acetate groups from incomplete PVA hydrolysis, although present at low levels, may deprotonate in alkaline conditions, further contributing to charge development. These combined effects have been experimentally observed in various PVA–oxide systems, where zeta potential measurements consistently show an increase in negative magnitude with rising pH.

The hydrodynamic diameter of these supernanostructures follows the expected trend that the higher the zeta potential (in absolute value), the smaller the structures/aggregates, mainly due to the introduction of interparticle electrostatic repulsion.^54^ TEM images of the supernanoparticles formed from the dissolution of the printlets at pH 6.8 corroborate this hypothesis, since the clusters resulting from the dissolution of the citrate-coated sample are smaller and more homogeneous than those from the bare Fe_3_O_4_ NPs sample (ζ_3D‑FeC_ > ζ_3D‑FeP_). Thus, the higher dissolution rate observed for the 3D-FeC sample can be correlated with the greater colloidal stabilization of the generated supernanoparticles, resulting from the ionization of carboxylic groups on the Fe_3_O_4_ NPs surface (≡Fe–COOH → ≡Fe–COO^–^), in contrast with the nonionized groups on the bare NPs (≡Fe–OH).

Studies on AgNPs^55^ have shown that digestive conditions promote aggregation and morphological changes, limiting their ability to cross the intestinal mucus and resulting in poor absorption. In contrast, our DLS and zetametry analyses revealed that citrate coating improves nanoparticle dispersion, particularly under alkaline conditions, due to the electrostatic stabilization arising from surface carboxyl deprotonation. This stabilization reduces aggregation and favors the formation of smaller, more homogeneous supernanostructures, which are better suited to penetrate the intestinal mucus and be internalized by enterocytes. The increased colloidal stability also provides a larger effective surface area for dissolution, supporting a more efficient release profile.^56^ Quantitatively, dissolution testing of the iron-loaded printlets confirmed pharmacopeial compliance, with over 80% of iron released within 60 min, but in a more gradual and sustained manner compared to ferrous sulfate tablets, which dissolve rapidly and often lead to supersaturation, precipitation, and reduced bioavailability.^57^

Overall, the dissolution results indicate that the superior performance of the 3D-FeC formulation is not limited to an initial kinetic effect but reflects a sustained and reproducible enhancement of iron release over time. This behavior is consistent with a formulation-dependent structure–property relationship, in which citrate functionalization improves colloidal stability, dispersion, and effective surface accessibility of the nanoparticles in the dissolution medium.

Although dissolution testing cannot fully replicate the complexity of physiological environments, the observed differences between formulations suggest potential relevance to in vivo release behavior. Future studies will explore in vitro–in vivo correlations to further refine the formulation design and evaluate the translational implications of these findings.

### FET Tests

According to Kimmel,^58^ the embryonic development of zebrafish progresses from 0.2 to 72 h until hatching. The test was monitored for 96 h, during which no significant lethal or sublethal effects were observed for the negative control. The maximum mortality observed was 1%, as recommended in the literature.

It was noted that different concentrations of the tested samples: (ii) bare and citrate-capped Fe_3_O_4_ NPs dispersions (respectively from FeP and FeC samples) and (ii) solutions from printlets (respectively from 3D-FeP and 3D-FeC dissolution) appeared homogeneous at the time of dissolution. Figure 7 provides an overview of the results of the embryotoxicity assay for Fe_3_O_4_ NPs in their various formulations and the impact fraction on embryonic development as a function of NP concentration (0.1–100 mg/L) over 24, 48, 72, and 96 h.

**Figure 7:**
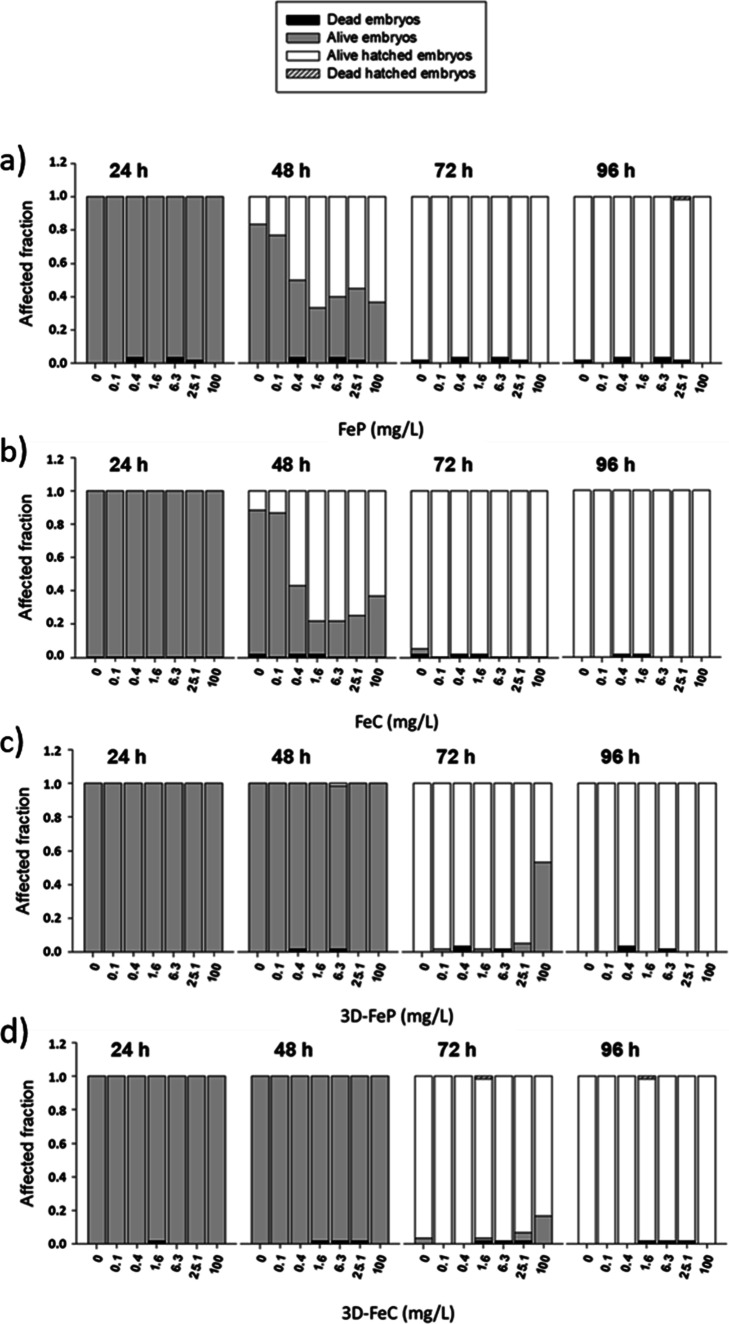
Affected fraction observed for suspensions of bare Fe_3_O_4_ nanoparticles (FeP) (a) and citrate-coated Fe_3_O_4_ nanoparticles (FeC) (b), and for suspensions obtained from the dissolution of printlets containing bare Fe_3_O_4_ nanoparticles (3D-FeP) (c) and citrate-coated Fe_3_O_4_ nanoparticles (3D-FeC) (d).

During the tests, a pattern of low embryotoxicity was observed; however, nanoparticles were observed adhering to the chorion of embryos exposed to the different test solutions (darkened chorion–Figure 8a), whereas the control group showed a normal chorion under optical microscopy (Figure 8d). After 72 h of exposure, hatching inhibition was observed in organisms exposed to 3D-FeP and 3D-FeC at 100 mg/L–approximately 50% and 10% of the embryos, respectively (Figure 7). This result was similar to that described in previous studies.^59,60^ Both authors demonstrated that, depending on nanoparticle size, interference with chorion can occur, partially or completely blocking its pores and limiting the exchange of nutrients and oxygen. Furthermore, the accumulation of nanoparticles on the chorion surface can delay embryonic development and/or alter hatching due to hypoxia and the generation of ROS.

**Figure 8:**
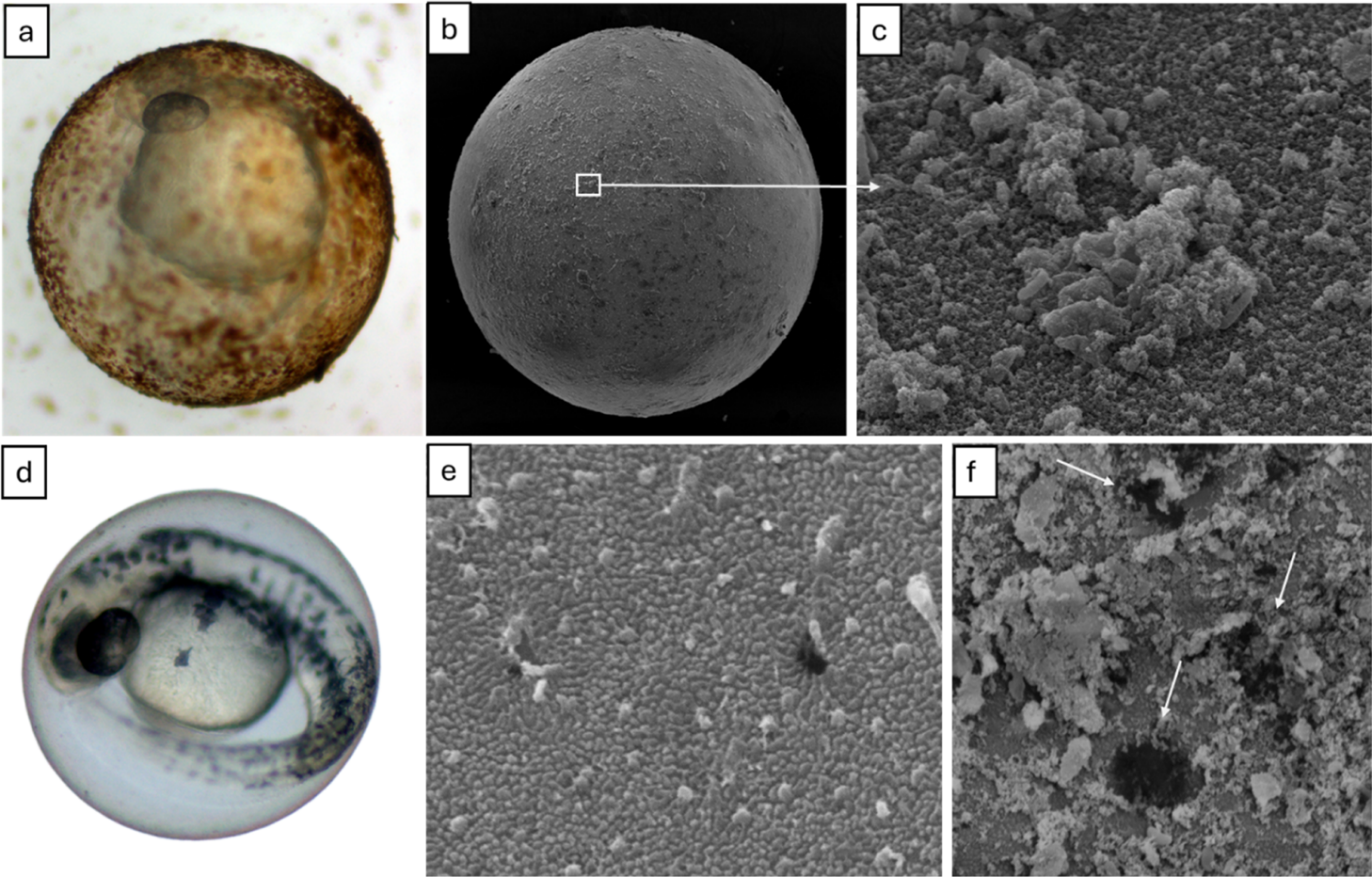
Images of optical microscopy (a) and SEM at 75× (b) and 5000× (c) of a zebrafish embryo exposed to sample 3D-FeP, showing Fe_3_O_4_ NPs distributed across the chorion surface. Optical microscopy image of a zebrafish embryo from the control group, without exposure to Fe_3_O_4_ NPs (d). SEM image of the chorion surface from the control group (no exposure to Fe_3_O_4_ NPs), at 5000× magnification (e). Chorion surface after exposure to Fe_3_O_4_ NPs, showing nanoparticles dispersed around the pores, as indicated by the arrows (f).

It is worth noting that, in the present study, the aforementioned effect was not observed the following day (96 h) in organisms exposed to the highest concentration (100 mg/L) (Figure 7c). However, variation in hatching time is not a definitive indicator of toxicity, as larvae can develop normally at later stages. The absence of changes in hatching behavior may indicate that iron nanoparticles that penetrated the chorion are accumulating in the organs. In this case, their toxic effects would manifest only in the final stages of zebrafish development.^61^

The SEM analyses, Figure 8b,c,e,f, show the surface of different chorions. Quadrant “*E*” – 0 mg/L (negative control), presents normal structural conformation and free pores. In quadrants C and F, agglomerated NPs are observed close to the pores.

At the end of the tests, the LC_50_ (96 h) for all treatments was above the highest concentration tested (100 mg/L), demonstrating low lethal toxicity of iron NPs FeP; FeC; 3D-FeP and 3D-FeC for zebrafish embryos, according to previous studies.^62,63^ It is worth noting that the observed sublethal effect (delay in hatching) may be related to the interference with some pores,^64,65^ as NPs were observed around, partially or fully covering the pores. Furthermore, embryos exposed to 3D-FeC showed less effect on hatching than those exposed to 3D-FeP. Several studies^59,66–69^ that have reported similar delayed or sublethal toxicity patterns in zebrafish have consistently shown low acute toxicity for iron oxide nanoparticles, including magnetite (Fe_3_O_4_), hematite (α-Fe_2_O_3_), and maghemite (γ-Fe_2_O_3_), with developmental effects typically observed only at relatively high exposure levels; additionally, surface functionalization approaches, such as citrate coating, have been associated with reduced biological impact in zebrafish models. This result indicates that citrate-functionalized iron NPs may be important in the development of biotechnological research.

## Conclusions

This work demonstrates the successful integration of iron oxide nanoparticles into three-dimensional printed dosage forms through hot-melt extrusion and fused deposition modeling, establishing a novel platform for advanced oral iron supplementation. Filaments prepared from stable glycerol-based nanoparticle dispersions exhibited superior mechanical properties and printability, ensuring homogeneous incorporation of both bare and citrate-coated Fe_3_O_4_ nanoparticles into the resulting printlets. Dissolution studies revealed that while both formulations achieved rapid release under gastric conditions, citrate-functionalized nanoparticles provided markedly enhanced iron release in enteric media, consistent with improved colloidal stabilization of the released nanostructures. Zebrafish embryo assays confirmed low acute toxicity across all formulations, with citrate coating mitigating sublethal effects at higher concentrations. Collectively, these findings highlight that nanoparticle surface chemistry and dispersion strategy critically influence the performance of 3D-printed dosage forms. By combining the intrinsic benefits of iron oxide nanoparticles with the structural flexibility of additive manufacturing, this approach enables pharmaceutically relevant customizable formulations exhibiting low acute toxicity, with the potential to address key limitations of conventional iron supplements. Future efforts should focus on establishing in vitro–in vivo correlations and on assessing clinical efficacy to accelerate the translation of this technology into personalized supplementation therapies.

## Supplementary Material



## Notes

Experimental design of the Fish Embryo Toxicity (FET) test (Figure S1). SEM/EDS analysis of selected areas from Figures 3f and 4e (Figure S2) (PDF)
