# Supplementary material for: Iron Oxide Nanoparticles Integrated in 3D-Printed Dosage Forms for Advanced Iron Supplementation
Source: ACS Nanosci Au. 2026 Feb 3;6(3):386–99. doi: 10.1021/acsnanoscienceau.5c00159 (PMC13281021; doi:10.1021/acsnanoscienceau.5c00159)
Supplement: Supplementary file 1 [file ng5c00159_si_001.pdf]

Supporting Information

Iron Oxide Nanoparticles Integrated in 3D-Printed Dosage Forms for Advanced Iron Supplementation

Mac-Kedson Medeiros Salviano Santos[a], Alexandre Silva Santos[b], Ariane Pandolfo Silveira[c], Diego Sousa-Moura[d], Idejan Padilha Gross[e], Ingrid Gracielle Martins da Silva[c], Luis Alexandre Muehlmann[a], Cesar Koppe Grisolia[e], Sebastião William da Silva[b], Sônia Nair Báo[c], Marcilio Cunha-Filho[f], Marcelo Henrique Sousa[a*]

[a] *Green Nanotechnology Group, University of Brasilia, CEP 72220-900, Brasilia-DF, Brazil.*
[b] *Optical Spectroscopy Laboratory, Institute of Physics, University of Brasilia, Brasília-DF, 70910-900, Brazil.*
[c] *Electron Microscopy Laboratory, Cell Biology Department, Institute of Biological Sciences, University of Brasilia, Brasília, Distrito Federal, 70910-900, Brazil.*
[d] *Laboratory of Nanobiotechnology, Department of Genetics and Morphology, Institute of Biological Sciences, University of Brasilia, Brasilia, Distrito Federal, 70910-900, Brazil.*
[e] *Laboratory of Genetic Toxicology, Department of Genetics and Morphology, Institute of Biological Sciences, University of Brasília, Brasília-DF 70910-900, Brazil*
[f] *Laboratory of Food, Drug, and Cosmetics (LTMAC), School of Health Sciences, University of Brasilia, 70.910-900 Brasília, DF, Brazil.*

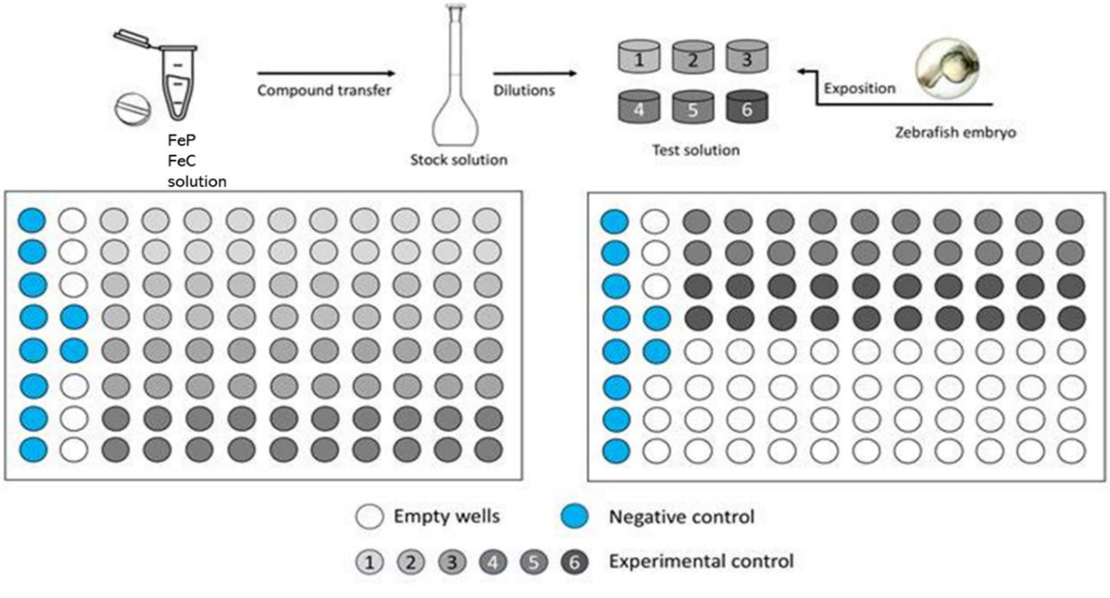

Figure S1 - Experimental design of Fish Embryo Toxicity (FET) test.

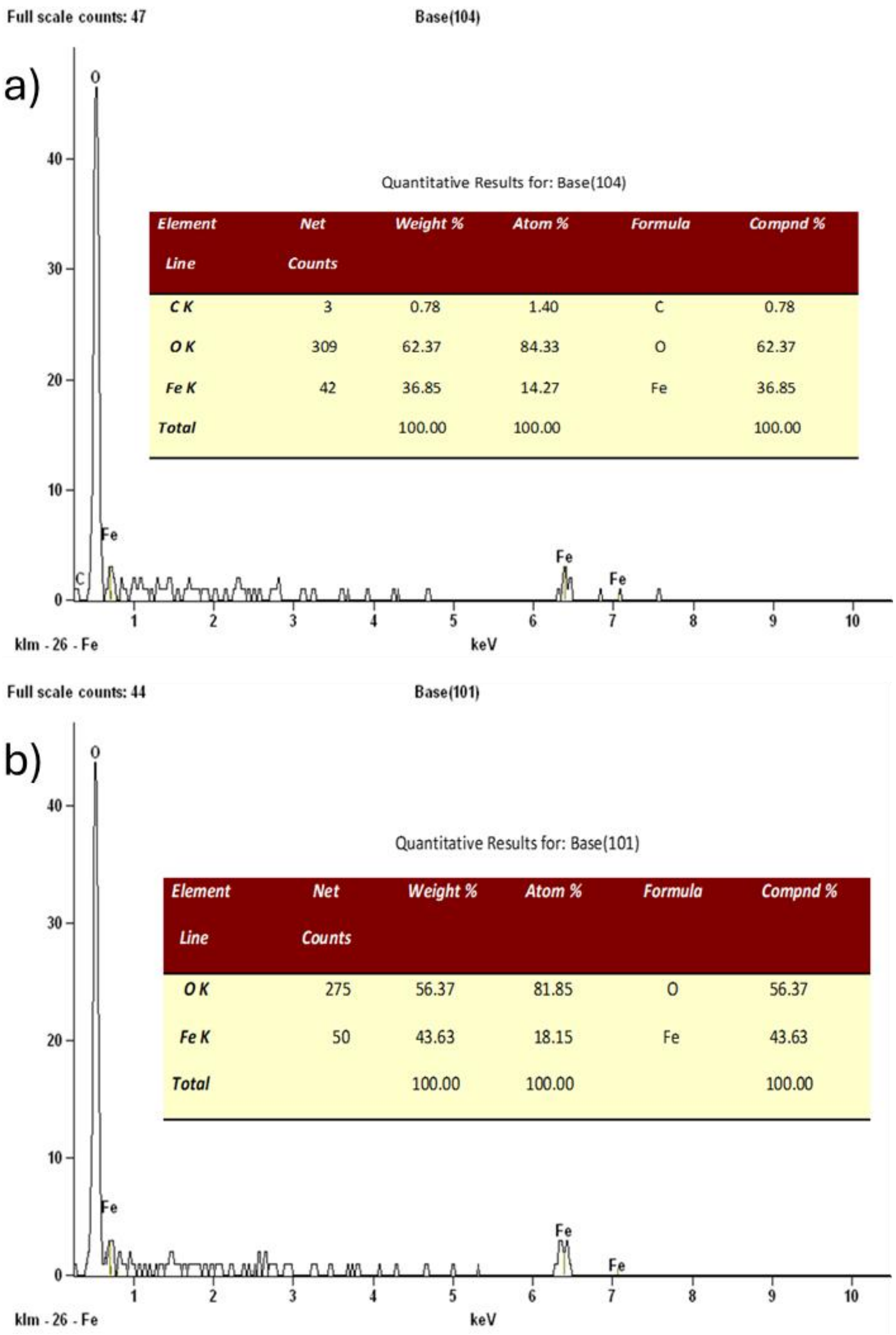

Quantitative Results for: Base(104)

Element Line	Net Counts	Weight %	Atom %	Formula	Compnd %
C K	3	0.78	1.40	C	0.78
O K	309	62.37	84.33	O	62.37
Fe K	42	36.85	14.27	Fe	36.85
Total		100.00	100.00		100.00

Quantitative Results for: Base(101)

Element Line	Net Counts	Weight %	Atom %	Formula	Compnd %
O K	275	56.37	81.85	O	56.37
Fe K	50	43.63	18.15	Fe	43.63
Total		100.00	100.00		100.00

Figure S2 – SEM/EDS analysis of selected areas shown in Figures 3f (a) and 4e (b).
